# Prognostic Factors and Survival in Patients with Radiation-Related Second Malignant Neoplasms Following Radiotherapy for Nasopharyngeal Carcinoma

**DOI:** 10.1371/journal.pone.0084586

**Published:** 2013-12-18

**Authors:** Mian Xi, Shi-Liang Liu, Lei Zhao, Jing-Xian Shen, Li Zhang, Peng Zhang, Meng-Zhong Liu

**Affiliations:** 1 State Key Laboratory of Oncology in Southern China, Guangzhou, China; 2 Department of Radiation Oncology, Cancer Center, Sun Yat-sen University, Guangzhou, China; 3 Collaborative Innovation Center of Cancer Medicine, Guangzhou, China; 4 Imaging Diagnosis and Interventional Center, Cancer Center, Sun Yat-sen University, Guangzhou, China; The University of Hong Kong, China

## Abstract

**Purpose:**

To analyze the clinicopathological characteristics, treatment modalities, and potential prognostic factors of radiation-related second malignant neoplasms (SMNs) in a large group of nasopharyngeal carcinoma (NPC) cases.

**Methods and Materials:**

Institutional electronic medical records of 39,118 patients with NPC treated by definitive radiotherapy between February 1964 and December 2003 were reviewed. A total of 247 patients with confirmed SMN attributable to radiotherapy were included.

**Results:**

Median latency between radiotherapy for NPC and the diagnosis of SMN was 9.5 years (range, 3.1–36.8 years). Squamous cell carcinoma was the most common histologic type, followed by fibrosarcoma and adenocarcinoma. Median progression-free survival and overall survival (OS) of the 235 patients who underwent treatment were 17.3 months and 28.5 months, respectively. The 5-year OS rates were 42.9%, 23.7%, and 0% for the surgery, radiotherapy, and chemotherapy groups, respectively. The independent prognostic factors associated with survival were sex, histologic type, and treatment modality in both the early stage subgroup and the advanced stage subgroup of SMN.

**Conclusions:**

Sex, histologic type, and treatment modality were the significant prognostic factors for SMN. Complete resection offers the best chance for long-term survival. In select patients with locally advanced and unresectable SMN, reirradiation should be strongly considered as a curative option.

## Introduction

Nasopharyngeal carcinoma (NPC) is a rare malignancy in most parts of the world, but it is one of the most common types of cancer in Southern China with an annual incidence of 25 to 50 cases per 100,000 persons [1]. Radical radiotherapy (RT) is the main treatment modality for NPC. Over the past 30 years, advances in the RT technique, imaging technology, and chemotherapy have led to a marked improvement in the survival of NPC patients. The preliminary reports of NPC treated with intensity-modulated RT (IMRT) showed excellent results with 3-year overall survival (OS) rates of 83.1% to 94.3% [2–4].

Ionizing radiation, a well-known carcinogenic agent, can induce malignancy [5,6]. With improved survival, the increasing incidences of second malignant neoplasms (SMNs) attributable to RT have become a major concern among long-term survivors. The incidence of SMN after NPC is substantially lesser than those of other head and neck tumors such as oral and laryngeal cancer [7–9]; this can be attributed to the unique predisposing factors of NPC. Because of the rarity of radiation-related SMN in NPC, few studies have addressed the therapeutic management and outcome of this disease. Moreover, the studies that have been performed on this topic are mainly on small series of patients or include a mix of different primary tumor entities, making it difficult to evaluate the optimal treatment strategy and prognostic factors [10–17]. In this article, we report on the analyses of the clinicopathological characteristics, treatment outcomes, and potential prognostic factors of SMN in a large group of NPC cases from endemic regions.

## Materials and Methods

### Eligibility

The inclusion criteria for radiation-related SMN were (1) prior history of RT; (2) second solid cancer situated within the previously irradiated field; (3) histologic confirmation of SMN, which is histologically different from the primary cancer; (4) a latency of at least 3 years between irradiation and SMN [18]. Squamous cell carcinomas (SCC) with different sites from the location of the primary NPC after more than a 5-year recurrence-free period were also included in the SMNs [19]. These criteria were modified from those for radiation-induced sarcoma, defined by Cahan et al. [20] and Arlen et al. [21].

Between February 1964 and December 2003, a total of 39,118 patients with NPC were treated by definitive RT in our Cancer Center. According to the institutional electronic medical records, 792 cases (581 males and 211 females) developed second solid cancers during the follow-up period (1967–2010). Within this cohort, 247 patients with radiation-related SMN who fulfilled the criteria were included. 

### Ethics statement

This study was approved by our Institutional Review Boards (IRBs) for Cancer Center, Sun Yat-sen University. Written informed consents were obtained from all adult patients in accordance with the regulations of IRBs. Written informed consents were obtained from the next of kin, caretakers, or guardians on the behalf of the minors/children participants involved in this study.

### Statistical Analysis

The cutoff date of the last follow-up was November 30, 2012 for the censored data analysis. Follow-up time was calculated from the time of diagnosis of SMN to the last date of contact. OS was calculated from the diagnosis of SMN until death or last follow-up. Progression-free survival (PFS) was measured until last follow-up, local, regional, or distant progression, or death. Kaplan-Meier method was used for analysis of OS and PFS; the log-rank test was used to examine the difference between groups; Cox regression model was used for multivariate analysis. The statistical analysis was performed using SPSS 13.0 software (SPSS Inc., Chicago, IL). A P value of < 0.05 was considered statistically significant.

## Results

### Patient Characteristics

The main characteristics of the patients at diagnosis of NPC are summarized in Table 1. Median age at NPC diagnosis was 43 years (range, 13–71 years), and the male to female ratio was 3:1. Patients treated for NPC before 1986 were staged according to the Changsha staging system, while patients treated after 1986 were restaged according to the 2002 American Joint Committee on Cancer (AJCC) staging system. The radiation techniques have been described in detail in the previous report [4,22]. Median doses to the nasopharyngeal region and neck region were 68.0 Gy (range, 54.2–92.0 Gy) and 53.6 Gy (range, 30.8–78.0 Gy), respectively. The daily fraction size was 2 Gy delivered using Cobalt-60 or 6-MV photons in most patients. Thirty-six patients received neoadjuvant or concurrent chemotherapy. Various chemotherapy regimens were used, which included cisplatin-based regimens in 29 patients, fluorouracil in 3 patients, and cyclophosphamide in 4 patients.

**Table 1:** Characteristics of patients and tumors at the time of nasopharyngeal carcinoma diagnosis (*n* = 247).

Characteristic	No. of patients	%
Sex		
Male	186	75.3
Female	61	24.7
Age at diagnosis of NPC (years)		
≤ 43	134	54.3
> 43	113	45.7
Calendar period at diagnosis of NPC		
1964-1975	44	17.8
1976-1985	59	23.9
1986-1995	67	27.1
1996-2003	77	31.2
Smoking		
Yes	105	42.5
No	142	57.5
Excessive alcohol intake		
Yes	11	4.5
No	236	95.5
Family history of cancer		
Yes	36	14.6
No	211	85.4
TNM Stage		
I and II	154	62.3
III and IV	93	37.7
Histologic type		
WHO type I/II	9	3.6
WHO type III	238	96.4
Radiation machine*		
Orthovoltage X-rays	24	9.7
Cobalt-60	183	74.1
Megavoltage X-rays	40	16.2
Radiation course		
Split course	135	54.7
Continuous course	112	45.3
Radiation technique		
Conventional radiotherapy	242	98.0
3DCRT	4	1.6
IMRT	1	0.4
Radiation dose to SMN region (Gy)		
≤ 68	141	57.1
> 68	106	42.9
Alkylating agents chemotherapy		
Yes	29	11.7
No	218	88.3

Abbreviations: NPC, nasopharyngeal carcinoma; WHO, World Health Organization; 3DCRT, three-dimensional conformal radiotherapy; IMRT, intensity-modulated radiotherapy; SMN, second malignant neoplasm; Gy, gray.

^*^ A radiation machine was used to irradiate the SMN region.

### Second Malignant Neoplasms

The crude incidence of SMN after definitive RT for NPC was approximately 0.63%. At the time of SMN diagnosis, the median age was 53 years (range, 18–77 years). Median latency between the start of RT for NPC and the diagnosis of SMN was 9.5 years (range, 3.1–36.8 years). Twenty-six (10.5%) patients developed SMN within 3-5 years after RT, 109 (44.1%) within 5-10 years, and 112 (45.3%) more than 10 years. The median latency was 7.1 years (95% confidence interval [CI], 5.3–9.0 years) in patients who underwent a RT dose of more than 68 Gy to the SMN region, whereas the median latency was 10.3 years (95% CI, 9.0–12.1 years; *P* = 0.006) in patients who received less than 68 Gy. Median latency time was 5.2 years (95% CI, 3.2–7.2 years), 8.0 years (95% CI, 5.3–10.8 years), and 10.6 years (95% CI, 5.3–16.0 years) for patients treated with megavoltage X-rays, Cobalt-60, and orthovoltage X-rays, respectively (*P* = 0.001). Median latency was shorter for patients who had received additional chemotherapy (6.4 years; 95% CI, 4.6–8.3 years) than for those who had received RT alone (9.8 years; 95% CI, 9.2–10.4 years; *P* = 0.012). Latency was not influenced by gender, age, smoking, alcohol intake, family history, TNM stage of NPC, radiation course (split or continuous), or histologic type of SMN.

The locations and histologic types of SMN are presented in Table 2. The three main sites of SMN were the tongue, nasal cavity/paranasal sinuses, and gingiva. SCC was the most common histologic type, followed by fibrosarcoma, adenocarcinoma, and osteosarcoma. Grading of SMN was possible for 231 patients and the tumors were classified as follows: grade 1 for 98 tumors, grade 2 for 30 tumors, and grade 3 for 103 tumors. At the time of SMN diagnosis, lymph node metastasis was detected in 30 patients, and distant metastasis was detected in 2 patients. The tumors were classified according to the 2002 AJCC staging as follows: stage I, 45 (18.2%) tumors; stage II, 101 (40.9%) tumors; stage III, 50 (20.2%) tumors; and stage IV, 51 (20.6%) tumors.

**Table 2:** Location and histologic types of second malignant neoplasms (*n* = 247).

Characteristic	No.	%
**Location of disease**		
***Oral cavity***	114	46.2
Tongue	58	23.5
Gingiva	35	14.2
Hard palate	15	6.1
Bucca cavioris	6	2.4
***Nasal cavity/paranasal sinuses***	44	17.8
***Pharynx and Larynx***	27	10.9
Larynx	7	2.8
Base of tongue	7	2.8
Soft palate	6	2.4
Tonsil	5	2.0
Hypopharynx	2	0.8
***Other sites***	62	25.1
Neck	17	6.9
Face	9	3.6
External auditory	9	3.6
Maxilla	7	2.8
Mandible	6	2.4
Thyroid	6	2.4
Salivary gland	5	2.0
Brain	3	1.2
**Histologic type**		
***Carcinoma***	186	75.3
Squamous cell carcinoma	163	66.0
Adenocarcinoma	15	6.1
Basal cell carcinoma	3	1.2
Malignant melanoma	2	0.8
Malignant mixed tumor	1	0.4
Esthesioneuroblastoma	1	0.4
Glioma	1	0.4
***Sarcoma***	61	24.7
Fibrosarcoma	25	10.1
Osteosarcoma	14	5.7
Malignant fibrous histiocytoma	9	3.6
Rhabdomyosarcoma	2	0.8
Neurofibrosarcoma	2	0.8
Malignant neurilermmoma	2	0.8
Angiosarcoma	2	0.8
Meningeal sarcoma	2	0.8
Carcinosarcoma	1	0.4
Chondrosarcoma	1	0.4
Myofibroblastoma	1	0.4

### Treatment of SMN

Of the 247 patients with SMN, 12 patients refused treatment, while 38 patients received only palliative chemotherapy due to unresectable lesions or rejection of surgery by the patients. The majority of patients underwent surgery: surgery alone in 113 patients, surgery plus chemotherapy in 16, and surgery plus RT with or without chemotherapy in 22. Thirty-two patients were treated with RT alone, and 14 patients were treated with combined RT and chemotherapy without surgery. 

Complete resection was performed in 121 (80.1%) of the 151 patients, while the remaining 30 patients (19.9%) had macroscopically positive surgical margins or gross residual disease. The median dose was 66 Gy (range, 40.0–82.0) for the 68 patients who underwent reirradiation. Of the 72 patients who received chemotherapy, the most common chemotherapeutic regimens were cisplatin + 5-fluorouracil + bleomycin (BPF) for carcinoma and doxorubicin + ifosfamide + dacarbazine + mesna (MAID) for sarcoma.

### Follow-up and Outcome

The median follow-up period for all patients was 26.9 months (range, 0.7–395.7 months). Of the 235 patients who underwent treatment, 63 patients (26.8%) developed recurrent disease (56 local failure and 7 regional failure), and 16 patients (6.8%) developed distant metastasis (7 lung, 7 bone, and 2 liver).The interval between curative treatment and the presentation of recurrence of the disease ranged from 1.8 to 208.0 months, with a median of 10.4 months.

Six patients (2.6%) developed the third primary neoplasms in head and neck. The median interval between the diagnosis of SMN and development of the third neoplasm was 4.7 years (range, 0.8–29.7 years). The anatomical sites of the third tumor were also within the previously irradiated fields i.e., the base of the tongue, neck, soft palate, hard palate, mandible, and maxillary sinus. The histologic types of the third tumor were squamous cell carcinoma in 3 patients, and basal cell carcinoma, osteosarcoma, and leiomyosarcoma in the remaining 3 patients.

**Figure 1:**
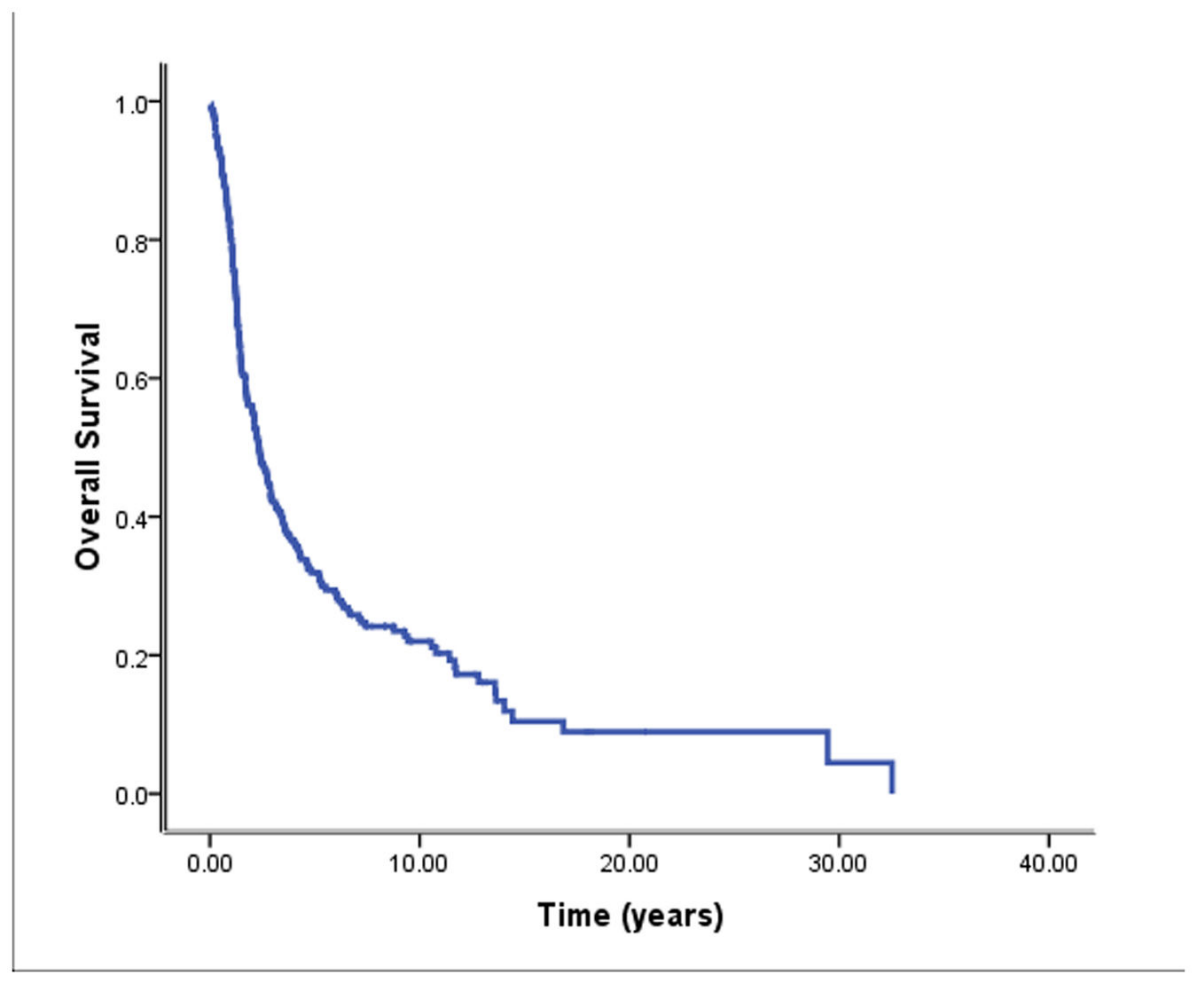
Overall survival in patients with radiation-related second malignant neoplasm (*n* = 235).

At the time of this analysis, 7 patients (3.0%) had been lost to follow-up, 186 patients (79.1%) had died, and 42 patients (17.9%) were still alive, including 28 patients without disease and 14 with disease progression. Median PFS and OS of the 235 patients under treatment were 17.3 months (95% CI, 13.4–21.2 months) and 28.5 months (95% CI, 23.0–34.1 months), respectively. The 5-year PFS and OS rates were 28.2% and 31.9%, respectively (Figure 1).

### Impact of Treatment Modality on Survival

**Figure 2:**
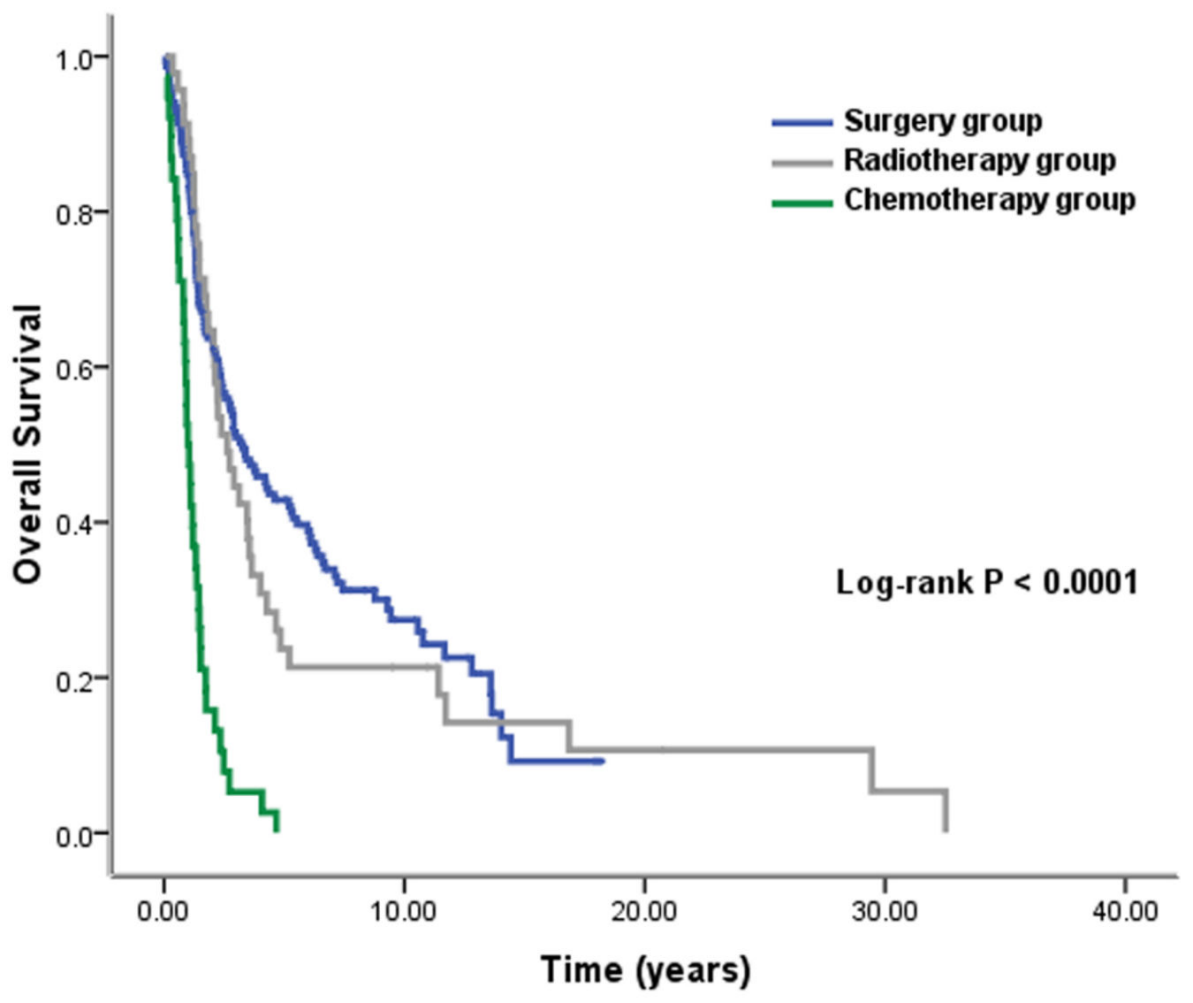
Overall survival according to treatment modalities of radiation-related second malignant neoplasm (*n* = 235).

Patients were classified into three groups according to treatment modalities: surgery (151 patients), RT (46 patients, including RT with or without chemotherapy), and chemotherapy (38 patients) groups. Five-year OS rates were 42.9%, 23.7%, and 0% in the surgery, RT, and chemotherapy groups, respectively (*P* < 0.0001, Figure 2). The median OS was 39.7 months (95% CI, 25.5–53.9 months) in the surgery group, 32.0 months (95% CI, 21.0–42.9 months) in the RT group, and 12.1 months (95% CI, 9.2–14.9 months) in patients who underwent palliative chemotherapy alone. 

The survival analysis of the surgery group yielded the following results: survival was significantly better in patients with complete resection than in those with incomplete resection (*P* < 0.0001); the median OS was 64.1 months (95% CI, 41.3–87.0 months) versus 10.8 months (95% CI, 5.8–15.8 months). For patients who underwent incomplete resection, no significant difference was detected in the survival between patients who underwent surgery alone and those who received combined therapy (*P* = 0.925).

### Prognostic Analysis of Survival in Subgroups

Because baseline patient characteristics may affect the selection of treatment modality, univariate and multivariate analysis were analyzed in subgroups with different TNM stage. Two subgroups were assessed: early TNM stage (I or II) and advanced TNM stage (III or IV). Univariate analysis revealed that survival was not affected by age at diagnosis of SMN, latency period, and the anatomic location of SMN in the two subgroups (Tables 3 and 4). The statistically significant or approaching significant factors in the univariate analysis were further analyzed by performing multivariate analysis. Multivariate analysis revealed that sex, histologic type, and treatment modality were the independent prognostic factors in both subgroups. However, the survival advantage of surgery over RT was not identical in the two subgroups. For early stage SMN, survival was significantly better in the surgery group than in the RT group (*P* = 0.013); Nevertheless, the RT group had similar survival outcome to the surgery group for the advanced stage patients (*P* = 0.731).

**Table 3:** Univariate and multivariate analysis for prognostic factors on survival in patients with early TNM stage (*n* = 141).

Factors	No.	5-Year OS (%)	Univariate *P*	Multivariate *P*
Sex				
Male	106	40.8	0.003	0.005
Female	35	68.8		
Age at NPC (years)				
≤ 43	83	41.3	0.886	
> 43	58	56.5		
Age at SMN (years)				
≤ 53	81	41.2	0.347	
> 53	60	56.0		
Latency (years)				
3-5	37	47.5	0.817	
5-10	40	41.1		
> 10	64	51.2		
Histologic type				
Carcinoma	105	53.0	0.002	0.048
Sarcoma	36	31.0		
Histologic grading				
Grade 1/2	75	60.7	< 0.0001	0.161
Grade 3	57	33.2		
Anatomic location				
Oral cavity	59	55.2	0.238	
Nasal cavity/paranasal sinuses	14	39.3		
Pharynx and larynx	19	47.2		
Others	49	39.7		
Treatment modality				
Surgery	106	55.5	< 0.0001	0.005
Radiotherapy	26	32.1		
Chemotherapy	9	0		

Abbreviations: OS, overall survival; NPC, nasopharyngeal carcinoma; SMN, second malignant neoplasm.

**Table 4:** Univariate and multivariate analysis for prognostic factors on survival in patients with advanced TNM stage (*n* = 94).

Factors	No.	5-Year OS (%)	Univariate *P*	Multivariate *P*
Sex				
Male	72	8.9	0.071	0.007
Female	22	9.1		
Age at NPC (years)				
≤ 43	48	6.4	0.025	0.111
> 43	46	11.4		
Age at SMN (years)				
≤ 53	45	5.0	0.843	
> 53	49	12.2		
Latency (years)				
3-5	19	6.3	0.581	
5-10	33	6.1		
> 10	42	12.3		
Histologic type				
Carcinoma	73	11.5	< 0.0001	< 0.0001
Sarcoma	21	0		
Histologic grading				
Grade 1/2	48	12.5	0.046	0.890
Grade 3	40	0		
Anatomic location				
Oral cavity	48	12.0	0.217	
Nasal cavity/paranasal sinuses	27	3.7		
Pharynx and larynx	6	16.7		
Others	13	7.7		
Treatment modality				
Surgery	45	13.7	0.003	< 0.0001
Radiotherapy	20	12.5		
Chemotherapy	29	0		

Abbreviations: OS, overall survival; NPC, nasopharyngeal carcinoma; SMN, second malignant neoplasm.

## Discussion

Exposure to ionizing radiation is a known risk factor for cancer. However, not all SMN that arise after RT are induced by irradiation, it is often difficult to differentiate radiation-related SMN from sporadic malignancies. In some types of tumors (lymphomas, breast cancer, etc.), the relative risk of SMN related to RT could be estimated in patients treated by RT versus patients treated by chemotherapy or surgery [6]. Nevertheless, this comparison cannot be performed in NPC patients. Therefore, we used the modified criteria for radiation-related SMN defined by previous reports [18–21].This is a report of the largest series published to date on the clinicopathological features, treatment outcomes, and related prognostic factors of SMN in patients with NPC; this investigation was conducted at a single institution from endemic regions over the past 40 years.

Approximately 10%–20% of patients with head and neck cancer have been reported to develop SMN after definitive therapy [9].The incidence of SMN after RT in patients with NPC ranged from 0.04% to 7% [10–13], significantly less than the high rates associated with other head and neck cancers. In this study, the crude incidence of SMN in NPC was estimated as 0.63%, comparable to the rate of 0.55% reported by Wang et al. [11]. The estimated incidence may be lower than the actual incidence because a proportion of patients were lost to follow-up or had died before the presentation of SMN. Additionally, the follow-up period for patients who received megavoltage X-rays is relatively short.

The median latency of 9.5 years in our series is consistent with the data from other studies, which ranged from 7.6 to 17.0 years [10–18]. Kuttesch et al. [23] reported that the administration of chemotherapy may shorten the latency between RT for the first cancer and subsequent presentation of SMN. This purported relationship between chemotherapy and latency was confirmed by our results. In addition, our results are consistent with previous studies suggesting that there may be a shorter latency interval in patients who receive higher radiation dose. Chen et al. [7] reported that the risk of sarcoma increased significantly after a latency period of some years and continued to increase after 10 years. We also found that nearly half of SMN occurred after a latency of more than 10 years, which indicated that a careful and adequately long-term follow-up protocol should be designed for patients with NPC. Additionally, the long-term follow-up should be maintained after curative therapy for SMN because a third neoplasm may occur over several years.

Amemiya et al. [19] reviewed the records of 1,358 patients treated by RT for head and neck SCC (HNSCC) and found that 25 patients developed SMN, including 18 tongue cancer and 3 maxillary sinus cancer. Scélo et al. [24] reported that tongue cancer significantly increased after radiation of NPC in a pooled analysis of 13 cancer registries (standardized incidence ratio = 11.1). In the study by Teshima et al. [25], the most common sites of SMN after NPC were oral cavity and pharynx. Consistent with previously published data, we noted that oral cancer was the most common type of SMN; particularly, malignancies in the tongue and gingiva accounted for approximately 40% of all SMN. Although a series of organs were exposed to high or intermediate dose of radiation, the incidences of developing SMN at different sites varied significantly. For example, SMN in thyroid, salivary gland, and brain were relatively rare. The most likely reason for this phenomenon is that the sensitivity of different organs to radio-induced carcinogenesis varies to a large extent [5].

The treatment outcome of SMN in head and neck was less satisfactory than that of patients with de novo primary head and neck cancer. The 5-year OS rate of 31.9% in our study was similar to that reported by Jones et al. [26], who found a 5-year OS rate of 31% in patients with second cancer following HNSCC. In a recent report from Taiwan, the 5-year OS rate was 30%, with the median survival of 1.7 years for 712 cases of SMN in NPC patients [7]. Their survival outcome is slightly lower than ours, primarily because the researchers included SMN of various regions, such as liver and lung cancers.

Our results indicated that SMN in the subtype of sarcoma was one of the independent prognostic factors associated with poor survival. Survival of sarcoma in our series approached the lower end of the previously reported wide range (range, 8%–60%) [27–29].The poor survival of patients with sarcoma can be attributed to several factors: pathologically aggressive features; difficulty of performing resection in the head and neck region; high potential for local recurrence and metastasis; insensitivity to chemotherapy and RT. The detailed report of prognostic factors for patients with sarcomas had been presented in the previous paper [30].

Despite the limited details of treatment modalities for SMN in the published reports, the important role of surgery has been documented in many reports [27–29]. In the current study, the 5-year OS rates of 55.5% obtained in the early stage subgroup are encouraging. Multivariate analysis revealed that surgical resection was one of the independent prognostic factors for better survival, which was in agreement with reports from Brady et al. [27] and Cha et al. [28]. Surgery for SMN in the head and neck region is rather challenging because of the difficulty of resection, high risk of surgical complications in a highly irradiated area, and the close proximity of the tumor to critical structures, such as carotid artery and skull base. However, compared with other treatment modalities, complete resection with a negative surgical margin apparently offers the best chance for long-term survival. With the median OS of only 10.8 months, the survival of the incomplete resection group was very poor. The current analysis showed that, in the incomplete resection group, survival after combined therapy was similar to that after surgery alone. However, the limited number of patients underwent incomplete resection did not permit us to reach a definitive conclusion in this regard.

Although surgery is preferable to RT as the treatment modality for SMN, often, resection cannot be performed in a substantial proportion of patients with locally advanced tumors. For unresectable SMN, treatment of chemotherapy alone is a palliative option with no long-term survivors in our series. The use of full-dose reirradiation for SMN is debatable. Theoretically, the tumor in the previously irradiated site could be insensitive or sometimes resistant to RT and chemotherapy, owing to tissue fibrosis after the first curative irradiation. Furthermore, the accumulation of a high radiation dose could result in serious organ injury. Nevertheless, the practice of reirradiation for second prmary head and neck cancer has been reported as feasible in recent years, particularly HNSCC [31–33]. As shown in our study, the survival of patients in the RT group was significantly better than that of patients who underwent chemotherapy alone, particularly in the carcinoma subgroup. In cases of patients with locally advanced and inoperable SMN, reirradiation with or without chemotherapy is the only possibility to offer the promise of long-term survival. Therefore, on the basis of histologic type of SMN, performance status, age, location, latency, tumor bulk, and pre-existing organ dysfunction, repeated RT should be strongly considered for treatment of a select proportion of patients. This being a long-term retrospective study with implying limitations, we did not deeply analyze the late complications of reirradiation in long-term survivors. Therefore, prospective studies with larger samples need to be performed to confirm this finding. Instead of two-dimensional RT technique, patients undergoing reirradiation should be treated with conformal techniques such as IMRT and proton-beam RT to limit the radiation dose to critical structures and to minimize the risk of complications.

In recent years, external RT technologies have evolved significantly, and IMRT has been increasingly used in the treatment of head and neck cancers. There is concern that IMRT might increase SMN risks because of the greater volume of normal tissues exposed to low-level dose of radiation [34]. We have used IMRT for treatment of a series of NPC patients at our institution since February 2001. At the time of this analysis, since only 1 patient of them had developed SMN, the effect of IMRT could not be assessed. Apparently, a longer follow-up and a larger series of patients will be necessary to confirm whether IMRT actually increases the incidence of SMN.

### Conclusions

Our study indicates that sex, histologic type, and treatment modality were independent prognostic factors for radiation-related SMN in NPC patients. Complete surgical resection apparently offers the best chance for long-term survival. Reirradiation should be strongly considered as a curative option in a select proportion of patients with locally advanced and unresectable SMN.
